# One-year mortality among hospital survivors of cholinesterase inhibitor poisoning based on Taiwan National Health Insurance Research Database from 2003 to 2012

**DOI:** 10.1186/s40360-018-0263-9

**Published:** 2018-11-13

**Authors:** Min-Chun Chuang, Chih-Hao Chang, Chung Shu Lee, Shih-Hong Li, Ching-Chung Hsiao, Yueh-Fu Fang, Meng-Jer Hsieh

**Affiliations:** 1 Department of Pulmonary and Critical Care Medicine, Chiayi Chang-Gung Memorial Hospital, No. 6, Sec. West, Chia-Pu Road, Pu-Tz City, Chiayi, 613 Taiwan; 2 Graduate Institute of Clinical Medical Sciences, College of Medicine, Chang Gung University, Taoyuan City, Taiwan; 3 Department of Pulmonary and Critical Care Medicine, Linkou Chang-Gung Memorial Hospital, Chang-Gung medical foundation, Taoyuan City, Taiwan; 4 Division of Pulmonary and Critical Care, Department of Internal Medicine, Saint Paul’s Hospital, Taoyuan City, Taiwan; 5 Chang-Gung University College of Medicine, Taoyuan City, Taiwan; 6 Department of Nephrology, Chang Gung Memorial Hospital, Taoyuan City, Taiwan; 7 Department of Respiratory Care, Chang-Gung University, Taoyuan City, Taiwan

**Keywords:** Organophosphates intoxication, Intensive care unit, Respiratory failure, Mechanical ventilation

## Abstract

**Background:**

Acute cholinesterase inhibitor (CI) poisoning, including organophosphate and carbamate poisoning, is a crucial problem in developing countries. Acute intoxication results in a cholinergic crisis, neurological symptoms, or respiratory failure. However, the short-term and long-term outcomes of CI poisoning are seldom reported.

**Methods:**

Data from the National Health Insurance Research Database were used to investigate the outcomes after organophosphate and carbamate poisoning. Patients who were hospitalized for a first episode of acute CI poisoning between 2003 and 2012 were enrolled in this study. Outcomes of acute CI poisoning with or without mechanical ventilation were analyzed.

**Results:**

Among 6832 patients with CI poisoning, 2010 developed respiratory failure requiring mechanical ventilation, and the other 4822 patients did not require mechanical ventilation. The hospital mortality rate was higher in patients requiring mechanical ventilation than in those not requiring mechanical ventilation (33.3% versus 4.7%, *p* < 0.0001). In patients with respiratory failure with mechanical ventilation, the patients without pneumonia had higher mortality rate than those with pneumonia. (36.0% versus 19.9%, *p* < 0.0001). The 1-year mortality rate the survivors of CI poisoning was 6.7%. Among 5932 survivors after cholinesterase inhibitor poisoning, the one-year mortality rate in patients with mechanical ventilation during hospitalization was higher than those without mechanical ventilation during hospitalization (11.4% versus 5.4% respectively, p < 0.0001).

**Conclusions:**

The one-year mortality rate of survivors after CI poisoning was 6.7%. Meanwhile, age, pneumonia, and mechanical ventilation may be predictive factors for the one-year mortality among the survivors after CI poisoning. Diabetes mellitus was not a risk factor for hospital mortality in patients with CI poisoning.

## Background

Organophosphates (OP) have been used as pesticides in countries where considerable agricultural activities are performed for more than 50 years. The acute intoxications caused by cholinesterase inhibitors (CI), including organophosphates and carbamates (CM), is a major problem, either by accidental ingestion or due to suicide attempts [1].

Both organophosphates and carbamates are potent cholinesterase inhibitors capable of causing severe cholinergic toxicity following cutaneous exposure, inhalation, or ingestion [2]. Cholinesterase inhibitors act by inhibiting acetylcholinesterase, which causes the accumulation of acetylcholine within synaptic clefts, resulting in the overstimulation and disruption of neurotransmission in both the central and peripheral nervous systems. The cholinergic overload leads to characteristic muscarinic, nicotinic, and central nervous system symptoms, including bradycardia, miosis, lacrimation, salivation, bronchorrhea, bronchospasm, urination, emesis, and diarrhea [3]. Respiratory failure following acute CI poisoning is a multi-factorial process; it includes depression of the central nervous system, neuromuscular weakness, excessive respiratory secretions, bronchoconstriction, and pneumonia [4, 5].

The standard therapeutic strategy for cholinesterase inhibitor poisoning includes decontamination, atropine, oximes, benzodiazepines and supportive care. Atropine and oximes are used to counteract the cholinergic symptoms, and benzodiazepines are used to prevent seizure activities [6]. However, the high mortality rate of acute CI poisoning ranges from 10 to 50%, even in the setting of highly sophisticated intensive care [7–12]. Few studies have reported the following outcome after organophosphate intoxication. In this study, we assessed the outcomes of OP and CM poisoning by using data from the National Health Insurance Research Database (NHIRD).

## Methods

### Ethics statement

The study protocol was approved by the Institutional Review Board of Chang-Gung Medical Foundation (No. 104-8058B). Research was conducted in accordance with the 1964 Declaration of Helsinki and its later amendments. All personally identifiable information was encrypted prior to the release of the Taiwanese NHIRD (https://nhird.nhri.org.tw/en/). Consequently, patient consent was waived for this study.

### Data source

This retrospective longitudinal study used data from the NHIRD, which is released and managed by the Taiwan National Health Research Institute. The National Health Insurance program is a mandatory social insurance program established by the Taiwanese government. It has provided comprehensive health care for all Taiwanese citizens since March 1, 1995, and currently covers 23.7 million enrollees, representing approximately 99.0% of the national population. The NHIRD comprises enrollment files, claims data, catastrophic illness files, and a drug prescription registry. It represents one of the largest nationwide healthcare service databases in the world.

### Patients and outcome

Patients who were hospitalized with a first diagnosis of OP and CM poisoning [International Classification of Diseases, Version 9, Clinical Modification (ICD-9-CM) code 989.3] between January 2003 and December 2012 were enrolled as the study subjects. The baseline comorbidities of the subjects were retrieved from the inpatient claims data. The following comorbidities were defined: diabetes mellitus (DM; ICD-9-CM code 250), hypertension (ICD-9-CM codes 401–405), coronary artery disease (ICD-9-CM codes 410–414), cerebrovascular accident (ICD-9-CM codes 430–438), asthma (ICD-9-CM codes 493), liver failure (ICD-9-CM codes 570), chronic obstructive pulmonary disease (ICD-9-CM codes 491, 492, and 496), and chronic renal failure (ICD-9-CM code 585). The exclusion criteria were 1) patients younger than 18 years (*n* = 89) and 2) subjects with missing data for sex (*n* = 7). All citizens in Taiwan are mandatory to participate in National Health Insurance program, and death was defined as the date of withdrawal from the insurance system according to the NHIRD data. In the previous study, the prognostic factors in organophosphate poisoning such as mechanical ventilation (MV) [10, 13], pneumonia [14], renal failure [9], and some medical comorbidities (diabetes mellitus, coronary artery disease, cerebrovascular accident, hypertension), [15] have been reported. These clinical variables were included in the analysis of mortality rate. Non-invasive ventilation was not included in the study.

### Statistical analysis

Data are expressed as mean ± standard deviation for continuous variables and as frequency and percentage for categorical variables. Chi-square tests were used for categorical variables, and Student t tests were used for continuous variables. Cox proportional hazard regression model was used to identify possible risk factors for mortality rate in patients with cholinesterase inhibitor poisoning intoxication. The variables used in the univariate COX proportional hazard regression model were age, sex, common comorbidities, medication, pneumonia, hemodialysis, and mechanical ventilation. The variables with *P* values < 0.05 in the univariate model were used in the multivariate analysis. Survival curves were plotted by using the Kaplan–Meier approach and were compared through the log rank test. All statistical analyses were performed by using MedCalc, version 12.5 (MedCalc Software, Ostend, Belgium). Two-tailed *p* values less than 0.05 were considered statistically significant.

## Results

Baseline characteristics of these patients with CI poisoning are shown in Table 1. From January 2003 to December 2012, 6832 patients were hospitalized with a diagnosis of CI intoxication. The mean age of the patients was 55.39 ± 16.44 years, and 2038 (29.3%) patients were female. Among these patients, 29.4% had respiratory failure and received mechanical ventilation during hospitalization. The overall hospital mortality rate in patients with OP and CM intoxication was 13.2%.

**Table 1:** Characteristics of patients with acute cholinesterase inhibitor poisoning (*N* = 6832)

Characteristics	
Age, mean ± SD, years old	55.39 ± 16.44
Female Sex, N (%)	2038 (29.8)
Comorbidities	
Diabetes mellitus, N (%)	773 (11.3)
Hypertension, N (%)	909 (13.3)
Coronary artery disease, N (%)	192 (2.8)
Cerebrovascular accident, N (%)	177 (2.6)
Chronic renal failure, N (%)	53 (0.8)
Chronic obstructive pulmonary disease, N (%)	111 (1.6)
Asthma, N (%)	45 (0.7)
Liver failure, N (%)	30 (0.4)
Interventions	
Atropine use, N (%)	3653 (53.5)
Pralidoxime use, N (%)	3479 (50.9)
Hemodialysis, N (%)	124 (1.8)
Mechanical ventilation, N (%)	2010 (29.4)
Pneumonia, N (%)	492 (7.2)
Hospital mortality, N (%)	900 (13.2)
Hospital days, mean ± SD	9.21 ± 11.60

Data are presented as mean ± SD or N (%)

Table 2 shows the demographic and clinical characteristics of the survivors versus nonsurvivors of OP and CM poisoning. Age, sex, and the comorbidities of hypertension, chronic renal failure, and hyperlipidemia were significantly different between the survivors and nonsurvivors. A higher percentage of the nonsurvivors were given with atropine, pralidoxime (PAM), hemodialysis, and mechanical ventilation than the survivors. The hospital days was higher in survivors than in non-survivors (9.61 ± 11.43 vs. 6.58 ± 12.31, < 0.0001).

**Table 2:** Comparison of clinical characteristics between survivors and non-survivors of cholinesterase inhibitor poisoning (*N* = 6832)

	Non-survivors (*N* = 900)	Survivors (*N* = 5932)	95% CI of difference between means or percentages	*P* value
Age, mean ± SD, years old	62.18 ± 15.48	54.36 ± 16.34	6.682 to 8.959	< 0.0001
Female Sex, N (%)	306 (34.0)	1732 (29.2)	1.49 to 8.2%	0.0033
Comorbidities				
Diabetes mellitus, N (%)	117(13.0)	656 (11.1)	−0.38 to 4.4%	0.0867
Hypertension, N (%)	78 (8.7)	831 (14.0)	3.09 to 7.28%	< 0.0001
Coronary artery disease, N (%)	17 (1.9)	175 (3.0)	−0.097 to 2.02%	0.0727
Cerebrovascular accident, N (%)	32 (3.6)	145 (2.4)	0.0045 to 2.68%	0.0506
Chronic renal failure, N (%)	20 (2.2)	33 (0.6)	0.71 to 2.8%	< 0.0001
Chronic obstructive pulmonary disease, N (%)	16 (1.8)	95 (1.6)	−0.64 to 1.34%	0.6967
Asthma, N (%)	3 (0.3)	42 (0.7)	−0.25 to 0.75%	0.195
Liver failure, N (%)	11 (1.2)	19 (0.3)	0.27 to 1.86%	< 0.0001
Atropine use, N (%)	698 (77.6)	2955 (49.8)	0.55 to 4.69%	< 0.0001
Pralidoxime use, N (%)	564 (62.7)	2915 (49.1)	10.1 to 17.02%	< 0.0001
Hemodialysis, N (%)	59 (6.6)	65 (1.1)	3.94 to 7.34%	< 0.0001
Mechanical ventilation, N (%)	671 (74.6)	1339 (22.6)	48.83 to 55.01%	< 0.0001
Pneumonia, N (%)	85 (9.4)	407 (6.9)	0.55 to 4.69%	0.0052
Hospital days, mean ± SD	6.58 ± 12.31	9.61 ± 11.43	−3.846 to −2.225	< 0.0001

*CI*, Confidence interval; Data are presented as mean ± SD or N (%)

Among 6832 patients with CI poisoning, 2010 patients developed respiratory failure requiring mechanical ventilation, and the other 4822 patients were did not require mechanical ventilation. The hospital mortality rate was higher in patients with mechanical ventilation than in those without mechanical ventilation (33.3% versus 4.7%, *p* < 0.0001; Table 3). Besides, patients with mechanical ventilation had more hospital days than patients without mechanical ventilation (15.42 ± 16.33 vs. 6.62 ± 7.54, < 0.0001).

**Table 3:** Comparison of the clinical characteristics in patients with or without mechanical ventilation (*N* = 6832)

	With mechanical ventilation (*N* = 2010)	Without mechanical ventilation (*N* = 4822)	95% CI of difference between means or percentages	*P* value
Age, mean ± SD, years old	58.37 ± 16.38	54.15 ± 16.31	3.376 to 5.076	< 0.0001
Female sex, N (%)	654 (32.5)	1384 (28.7)	1.38 to 6.25%	0.0016
Diabetes mellitus, N (%)	259 (12.9)	514 (10.7)	0.5 to 3.97%	0.0081
Hypertension, N (%)	205 (10.2)	704 (14.6)	2.68 to 6.05%	< 0.0001
Coronary artery disease, N (%)	59 (2.9)	133 (2.8)	−0.75 to 1.04%	0.6865
Cerebrovascular accident, N (%)	76 (3.8)	101 (2.1)	0.79 to 2.71%	0.0001
Chronic renal failure, N (%)	24 (1.2)	29 (0.6)	0.098 to 1.21%	0.0110
Chronic obstructive pulmonary disease, N (%)	29 (1.4)	82 (1.7)	−0.41 to 0.92%	0.4426
Asthma, N (%)	13 (0.6)	32 (0.7)	−0.39 to 0.5%	0.937
Liver failure, N (%)	13 (0.6)	17 (0.4)	−0.16 to 0.67%	0.094
Atropine use, N (%)	1577 (78.5)	2076 (43.1)	33.06 to 37.67%	< 0.0001
Pralidoxime use N (%)	1453 (72.3)	2026 (42.0)	27.84 to 32.7%	< 0.0001
Hemodialysis, N (%)	83 (4.1)	41 (0.9)	2.32 to 4.19%	< 0.0001
Pneumonia, N (%)	322 (16)	170 (3.5)	10.83 to 14.25%	< 0.0001
Hospital mortality rate, N (%)	671 (33.4)	229 (4.7)	26.54 to 30.89%	< 0.0001
Hospital days, mean ± SD	15.42 ± 16.33	6.62 ± 7.54	8.224 to 9.358	< 0.0001

*CI*, Confidence interval; Data are presented as mean ± SD or N (%)

Table 4 shows the results for the univariate and multivariate analysis of variables associated with hospital mortality in patients with CI intoxication. Age, liver failure, atropine, hemodialysis, and mechanical ventilation were positively correlated with the hospital mortality rate. But hypertension, pneumonia, and PAM use were also negatively correlated with the hospital mortality.

**Table 4:** Clinical variables associated with hospital mortality in patients with organophosphate and carbamate poisoning analyzed by multivariate Cox regression (*N* = 6832)

Parameter	Univariate analysis	*P* value	Multivariate analysis	*P* value
	HR (95% CI)		HR (95% CI)	
Age	1.022 (1.018–1.026)	< 0.0001	1.020 (1.015–1.024)	< 0.0001
Female Sex	1.148 (0.999–1.318)	0.0506		
Diabetes	1.076 (0.885–1.306)	0.4635		
Hypertension	0.629 (0.497–0.794)	< 0.0001	0.615 (0.485–0.779)	0.0001
Coronary artery disease	0.582 (0.360–0.941)	0.0273	0.485 (0.300–0.785)	0.0751
Cerebrovascular accident	1.179 (0.828–1.679)	0.3607		
Chronic renal failure	2.634 (1.690–4.105)	< 0.0001	1.449 (0.893–2.351)	0.1330
Chronic obstructive pulmonary disease	0.875 (0.534–1.436)	0.5980		
Asthma	0.544 (0.175–1.688)	0.2920		
Liver failure	2.693 (1.486–4.882)	0.0011	2.348 (1.293–4.264)	0.0050
Atropine	2.406 (2.054–2.818)	< 0.0001	1.951 (1.628–2.337)	< 0.0001
Pralidoxime	1.184 (1.032–1.359)	0.0161	0.591 (0.506–0.691)	< 0.0001
Hemodialysis	2.520 (1.929–3.291)	< 0.0001	1.454 (1.087–1.946)	0.0117
Pneumonia	0.754 (0.600–0.947)	0.0154	0.480 (0.382–0.603)	< 0.0001
Mechanical ventilation	5.160 (4.426–6.014)	< 0.0001	4.684 (3.976–5.517)	< 0.0001

*CI*, Confidence interval; Data are presented as mean ± SD or N (%)

In Table 5, we applied univariate analysis to predict one-year mortality in survivors after CI poisoning. According to univariate analysis, age, diabetes mellitus, PAM use, hemodialysis, mechanical ventilation, and pneumonia during hospitalization were significant predictors of one-year mortality in patients with CI poisoning. After adjusting for these factors in a multivariate Cox regression analysis. Age, pneumonia, and mechanical ventilation were independent risk factors for one-year mortality among survivors of cholinesterase inhibitor poisoning.

**Table 5:** Clinical variables associated with one-year mortality in patients with organophosphate and carbamate poisoning analyzed by multivariate Cox regression (*N* = 5932)

Parameter	Univariate analysis	*P* value	Multivariate analysis	*P* value
	HR (95% CI)		HR (95% CI)	
Age	1.019 (1.013–1.026)	< 0.0001	1.016 (1.009–1.022)	< 0.0001
Female Sex	0.844 (0.674–1.056)	0.1385		
Diabetes mellitus	1.335 (1.006–1.771)	0.0451	1.185 (0.891–1.575)	0.2437
Hypertension	0.962 (0.722–1.282)	0.7923		
Coronary artery disease	1.110 (0.639–1.930)	0.7110		
Cerebrovascular accident	1.026 (0.548–1.923)	0.9360		
Chronic renal failure	1.932 (0.722–5.174)	0.1900		
Chronic obstructive pulmonary disease	1.293 (0.642–2.604)	0.4722		
Asthma	1.071 (0.344–3.337)	0.9062		
Liver failure	0.796 (0.112–5.658)	0.8194		
Atropine	0.992 (0.814–1.207)	0.9321		
Pralidoxime	1.367 (1.121–1.668)	0.0020	1.102 (0.892–1.362)	0.3669
Hemodialysis	2.443 (1.304–4.576)	0.0053	1.678 (0.890–3.163)	0.1095
Pneumonia	2.860 (2.196–3.725)	< 0.0001	1.962 (1.477–2.605)	< 0.0001
Mechanical ventilation	2.199 (1.796–2.692)	< 0.0001	1.722 (1.371–2.162)	< 0.0001

*CI*, Confidence interval; Data are presented as mean ± SD or N (%)

**Fig. 1:**
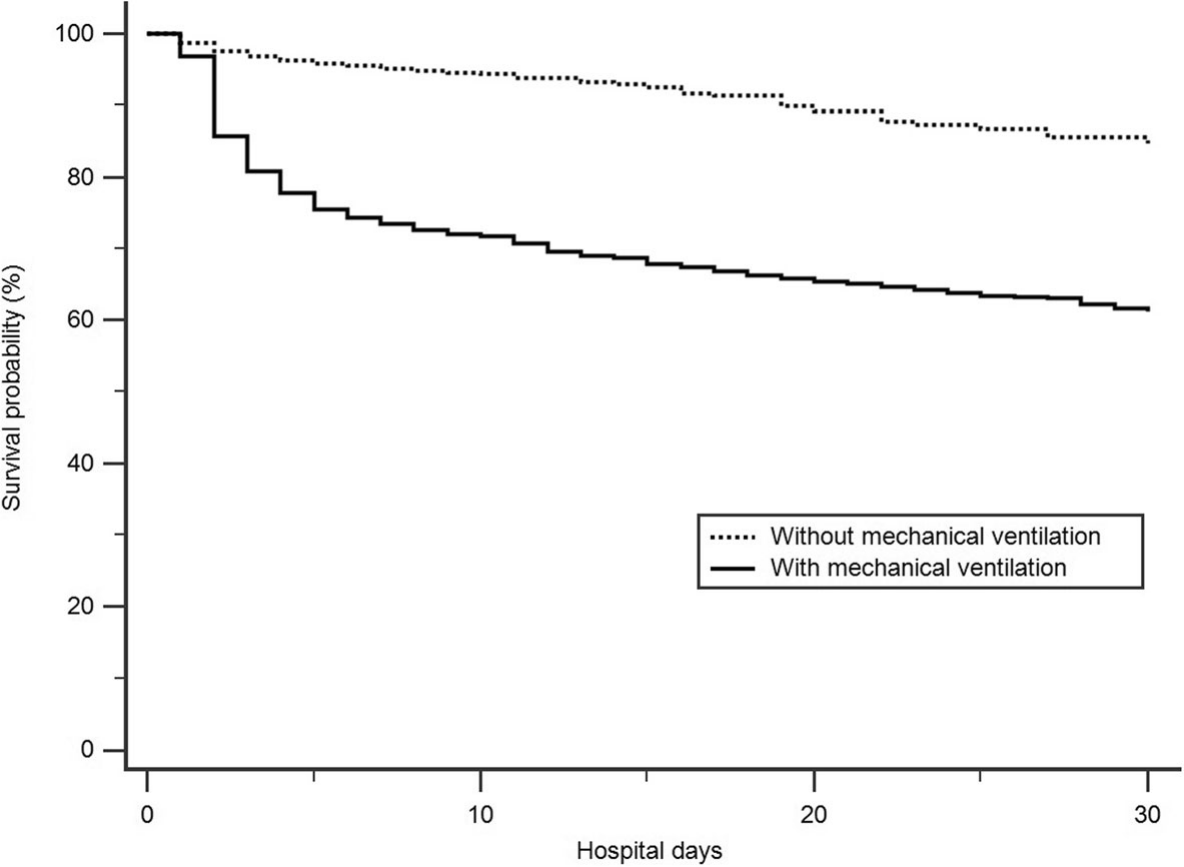
Kaplan-Meier survival curve for the patients with mechanical ventilation and without mechanical ventilation during hospitalization (*N* = 6832, Logrank test *P* < 0.0001)

Figure 1 illustrates the Kaplan–Meier survival curves indicating that the patients with OP intoxication who underwent mechanical ventilation exhibited a significantly higher hospital mortality rate (*p* < 0.0001) than did those who did not undergo mechanical ventilation.

**Fig. 2:**
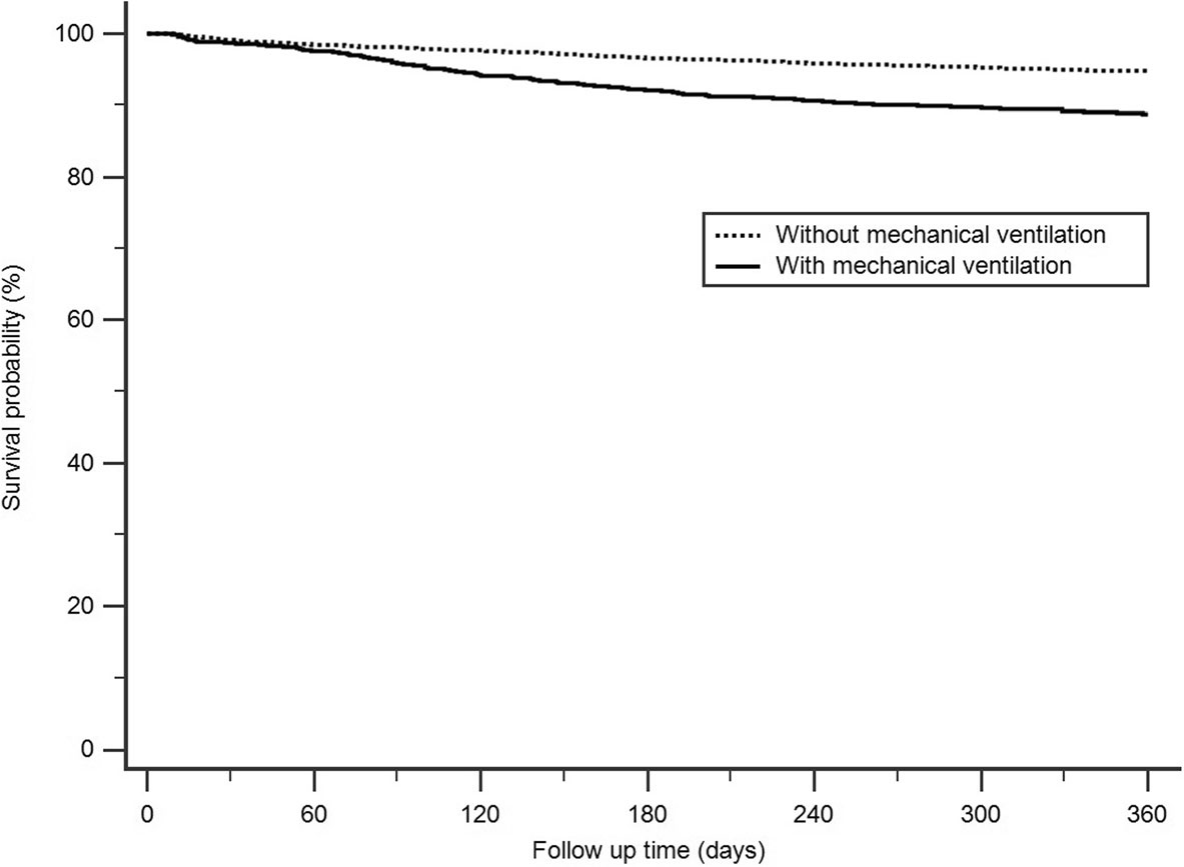
Kaplan-Meier survival curve of the survivors after organophosphate and carbamate poisoning, one year follow up (*N* = 5932, logrank test, P < 0.0001)

The 1-year survival rate of the survivors of CI poisoning was 93.3%. Among the 5932 survivors of CI poisoning, 1339 (22.6%) underwent mechanical ventilation during hospitalization and 152 (11.4%) died within 1 year. By contrast, 246 (5.4%) of the remaining 4593 survivors who did not require mechanical ventilation died within 1 year. Figure 2 shows that the 1-year cumulative survival rates differed significantly between survivors who underwent mechanical ventilation and those who did not undergo mechanical ventilation during admission (p < 0.0001).

## Discussion

This is the first nationwide population-based study with a relatively large number to investigate the one-year mortality of survivors after CI poisoning. Chronic renal failure, hemodialysis, and respiratory failure requiring mechanical ventilation were significantly associated with a poor outcome. For those survivors after CI poisoning, the one-year mortality rate of survivors after CI poisoning was 6.7%, and age, pneumonia, and mechanical ventilation were independent risk factors for one-year mortality among survivors of cholinesterase inhibitor poisoning. A recent prospective population-based cohort study in Unite State showed hospital mortality of pneumonia was 6.5%, but the one-year mortality was up to 30.6%. [16] The result is similar in our study that pneumonia is a predictive factor for the one-year mortality after CI poisoning.

Several strengths of this study are worth highlighting. First, this was a nationwide population-based study, meaning that a relatively large number of CI intoxication patients were included, and this sample was considered representative of the general population. Second, the study cohort was largely selected from a computerized database comprising all Taiwanese CI intoxication patients diagnosed between January 1, 2003, and December 31, 2012, thus decreasing the potential for selection bias.

The disease burden of OP and CM toxicity differs between Taiwan and most western countries [17]. Because OP and CM are easily accessed and highly lethal, this type of toxicity is one of the most frequent causes of poisoning in Taiwan [18]. Previous epidemiologic data of 1985–2006 indicate that most cases of OP exposure involve a single OP (80.37%), and the overall mortality rate is 12.71% [8], which is similar to that (13.2%) in our study.

Respiratory failure is one of the most important complications of CI poisoning. This may be due to respiratory muscle weakness, aspiration, hypersecretion, pneumonia, sepsis, or acute respiratory distress syndrome. [4]. A retrospective study of 155 patients reported that 59% of patients with CI poisoning developed respiratory failure [14]. In our study, 29.4% of patients had respiratory failure with mechanical ventilation. The mortality rate was 33.4% among the patients who were mechanically ventilated, whereas it was 4.7% among the patients who were not mechanically ventilated. (95% CI of the difference between percentages was 26.54 to 30.89%, *p* < 0.0001) These findings demonstrate that CI intoxication patients who require mechanical intubation had a higher mortality rate than those without mechanical intubation. This information aids clinical physicians in realizing that respiratory failure is a useful prognostic indicator in patients with CI poisoning.

In the previous reports, several prognostic factors were associated with CI poisoning, including age, serum cholinesterase and bicarbonate levels, and acute physiology and chronic health evaluation II scores [12, 19]. A retrospective study of 118 patients with OP poisoning reported that the DM status did not increase mortality [20]. This result is consistent with our findings that diabetes is not associated with both hospital mortality and one-year mortality. Sungur revealed that among patients with OP insecticide poisoning, the mortality rate was 50% in patients requiring mechanical ventilation and 21% in those not mechanically ventilated [11]. In the current nationwide study of 6832 patients, the hospital mortality rate was 33.4% among those with mechanical ventilation. A recent study demonstrated that acute renal failure was associated with mortality in organophosphorus poisoning. [9]. Our study showed that when patients with CI poisoning received hemodialysis during hospitalization, the hospital mortality rate was higher (OR; 6.332, 95% confidence interval: 4.419–9.074).

In our study, the prescription percentage of PAM and atropine was significantly higher in nonsurvivors than in survivors. This could be due to one of the two following reasons. First, the survivors may have experienced less CI toxicity and may have had less severe clinical symptoms. Therefore, PAM and atropine were not prescribed, according to clinical judgement. Second, most of the survivors may have experienced CM intoxication. Because PAM is not suggested for use in CM intoxication, the percentage of PAM use may have been lower in the survivors.

The findings of this study should also be interpreted in light of its limitations. First, serum laboratory data were not available in the NHIRD. Thus, we could not obtain the level of acetylcholinesterase activity, which is a direct biomarker for toxicity of organophosphorus. However, not all hospitals check acetylcholinesterase activity to the patients. Second, we could not categorize the precise intensity of the intoxication because NHIRD may not provide the detailed clinical course, such as clinical symptoms, the cause of intoxication (exposure to organophosphate or carbamates), the dose of toxin, the dose and length of antidote, or time to doctors. Third, the present study is a retrospective cohort study. Despite the meticulous design and control of some confounding factors, biases could remain because of possibly unmeasured or unknown confounding factors. We were unable to consider the severity of the diseases, which reduced our chances of showing the severity related effects of comorbidities. Finally, causes of death for the patients cannot be reported in the study because we have no death certificate databases.

## Conclusions

Among the survivors of CI poisoning, the one-year mortality rate was 6.7%. For the survivors after CI poisoning, age, pneumonia, and mechanical ventilation may be predictive factors for the one-year mortality. In addition, DM was not a risk factor for death in patients with CI poisoning.

## Abbreviations

CAD: coronary artery disease
CI: cholinesterase inhibitor
CM: carbamates
CNS: central nervous system
COPD: chronic obstructive pulmonary disease
CVA: cerebrovascular accident
DM: diabetes mellitus
NHIRD: National Health Insurance Research Database
OP: organophosphate
PAM: pralidoxime
